# A Giant Nevus Lipomatosus Cutaneous Superficialis Treated With Serial Surgical Excision: A Case Report

**DOI:** 10.7759/cureus.75054

**Published:** 2024-12-03

**Authors:** Aishwarya Pakhan, Sudhir Singh, Mayur Dudhe, Shweta Patil

**Affiliations:** 1 Dermatology, Datta Meghe Medical College, Nagpur, IND

**Keywords:** atypical, connective tissue, dermatology, nevus lipomatosus cutaneous superficialis, skin tumor

## Abstract

Nevus lipomatosus cutaneous superficialis (NLCS) is a rare dermatological condition characterized by ectopic mature adipocytes in the dermis. The classic variety presents multiple clustered skin-colored nodules, while the solitary form is a single papule or nodule. We report the case of a 25-year-old female who presented with multiple cerebriform nodules coalescing into a large plaque over the left iliac region that developed over 17 months. Lesions ranged from 0.5 cm to 6 cm in diameter. Histopathology confirmed classical NLCS. Treatment options were discussed, and serial surgical excision was opted for for a better cosmetic outcome. The largest nodule was excised under local anesthesia, while the remaining were removed six months later under short general anesthesia as daycare surgery. At the six-month follow-up, only a hypopigmented patch persisted at the surgery site. Serial excision allowed staged removal of this large classical NLCS for an excellent cosmetic result. Early diagnosis and conservative treatment prevent disfigurement and morbidity in this rare condition. Clinicians must recognize NLCS since therapeutic choices are restricted for widespread lesions of the classical subtype.

## Introduction

Giant nevus lipomatosus cutaneous superficialis (NLCS) is an uncommon benign skin condition encountered occasionally in clinical practice. It is marked by the abnormal deposition of fat within the dermal layer, presenting as soft, skin-colored, or slightly hyperpigmented nodules [1]. In the case of the giant variant, these lesions can extend over large areas, often causing significant cosmetic concerns for patients. Although benign, this condition can appear alarming due to the size of the lesion and its unusual appearance, leading many patients to seek dermatological consultation to understand their treatment options and alleviate anxieties about the possibility of malignancy [2].

Clinically, giant NLCS usually appears in areas like the buttocks, pelvic girdle, thighs, or lower back, though other body regions can be affected as well. The lesions are soft and feel fatty to the touch, often forming irregular, coalescing plaques that may extend over large sections of the skin [3]. Although most patients report no discomfort, some experience occasional itching or irritation. The condition is often asymptomatic, but its aesthetic impact can lead to emotional distress, especially when the lesion becomes extensive or noticeable [4]. Giant nevus lipomatosus cutaneous superficialis typically develops in early childhood or adolescence and, once formed, tends to remain stable without any significant changes over time [5]. Histologically, the presence of mature fat cells interspersed with collagen fibers within the dermis is the defining feature, helping differentiate it from other skin conditions [6].

Treatment for giant NLCS is generally driven by the patient’s cosmetic concerns rather than medical necessity, as the condition itself is harmless. The primary therapeutic option for larger lesions is surgical excision, which is often effective in completely removing the nevus [7]. However, in cases where the lesion is particularly large, a staged surgical approach might be required to minimize scarring. For smaller or less prominent lesions, less invasive techniques such as cryotherapy or laser therapy may be considered, although these methods are less definitive in terms of removing the entire lesion [8]. Liposuction can sometimes be used to reduce the volume of fatty tissue, particularly in lesions that are bulky but not necessarily harmful [9].

While the recurrence of giant NLCS after treatment is rare, monitoring the treated area post-procedure is important to ensure complete removal [1]. Patients are generally satisfied with the outcomes of surgery, as the risk of recurrence is low and cosmetic improvement is often significant [10]. Overall, the approach to treating giant NLCS focuses on understanding the patient’s concerns, educating them about the benign nature of the condition, and providing the best possible cosmetic outcomes through individualized treatment strategies [11]. This case report aims to present a rare case of giant NLCS in a young female, emphasizing its clinical presentation and treatment approach. This report highlights the successful use of serial surgical excision for achieving optimal cosmetic outcomes, demonstrating the importance of early diagnosis and tailored treatment in preventing disfigurement and minimizing morbidity.

## Case presentation

**Figure 1:**
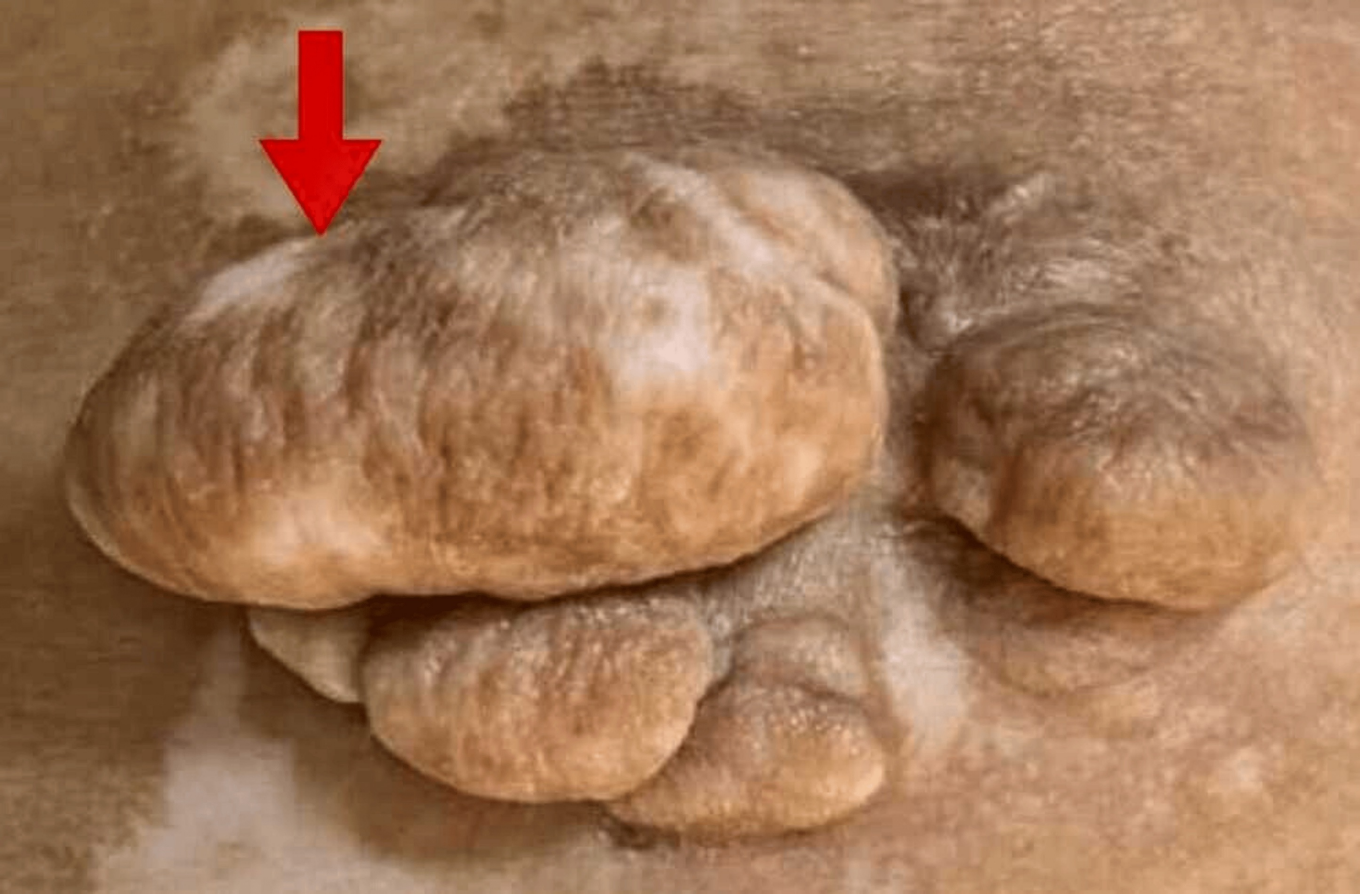
Multiple skin-colored nodules with depigmented base on the left flank The red arrow points to the single skin-colored large nodule with multiple skin-colored nodules with a depigmented base present over the left flank.

At the outpatient dermatology department, a 25-year-old healthy unmarried female with no relevant medical history presented with asymptomatic lesions that first appeared over her left flank around 17 months prior as small pea-sized skin-colored nodules that gradually increased in number and size, with the largest lesion being the size of an ivy gourd and smaller pea-sized lesions surrounding it. Three to four light-colored flat lesions were present around the main lesion (Figure 1). The lesion had an aesthetically unfavorable appearance. There were no similar complaints from family members.

On physical examination, there were six skin-colored, non-tender, mobile, well-defined pedunculated masses with overlying patches of hypopigmentation, firm in consistency, with cerebriform surfaces of varying sizes measuring from 0.5 cm to 1 cm in diameter. The largest measuring 3 cm x 2 cm was located over the left lower iliac region with a hypopigmented patch measuring 4 cm x 5 cm in the periphery, which had gradually increased to the present size. There was no history of minor trauma or irritation despite being in a friction-prone area. There was no hair growth, ulceration, discharge, café-au-lait macules, or induration. The mucous membranes, hair, nails, and scalp examination all revealed no abnormalities. A systemic assessment revealed nothing unusual. The patient was anxious about the cosmetic appearance of the lesion. She was informed about the benign nature of the lesion, and various treatment options were described to her. She opted for surgical excision. The patient was scheduled for surgery. Routine investigations were within the normal limit. She received clearance from the anesthesia department for the surgery.

**Figure 2:**
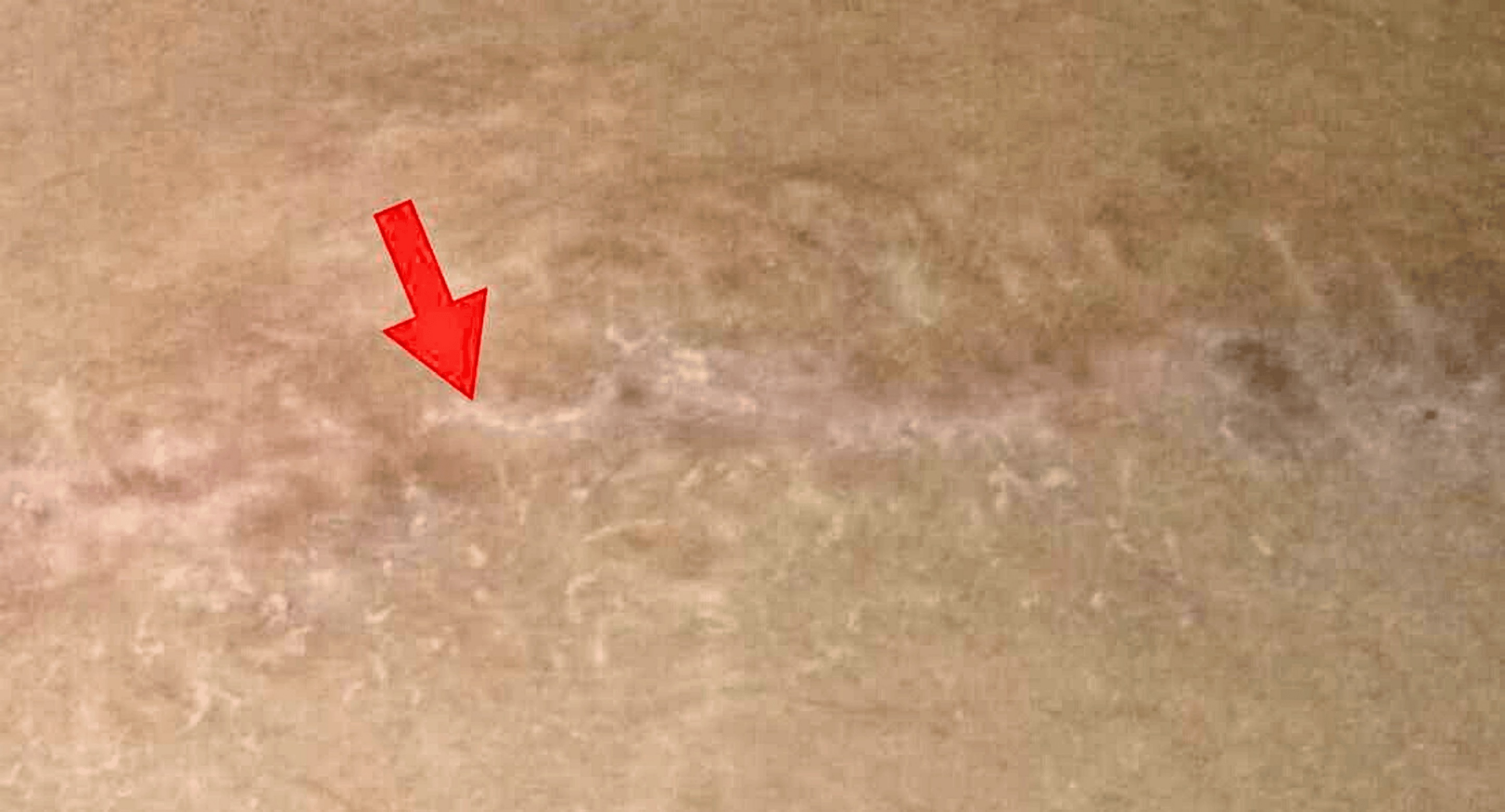
The red arrow shows the linear hypopigmented patch at the sixth-month follow-up

Serial excision of the lesion was planned for a better cosmetic outcome as the involved area was more than 7 cm in size and could not be excised in a single sitting. In the first sitting, the largest lesion was excised with an elliptical incision under local anesthesia, and the skin was sutured in two layers. The excised lesion was sent for histopathological examination, which revealed mature ectopic adipocytes among collagen fibers in the dermis. Hence, the diagnosis of NLCS was confirmed. The second sitting was done after six months, and all nodular lesions were excised under short general anesthesia as a daycare surgery. Hemostasis was achieved, and the skin was closed using continuous sutures with aseptic precautions. After the procedure, topical 2% mupirocin ointment was applied over the wound. At the sixth-month follow-up appointment, the patient reported no symptoms, and the examination revealed only a hypopigmented patch (Figure 2).

## Discussion

Nevus lipomatosus cutaneous superficialis is a rare form of idiopathic hamartomatous abnormalities that may start in infancy or be present at birth but can appear later in life [1]. Our patient's lesion is clinically and histologically compatible with NLCS. The clustered skin-colored to yellow-colored papules or nodules are frequently seen over the buttocks and lower back, especially in young patients, i.e., from birth to 20 years of age.

Two clinical subtypes have been identified for NLCS, namely the classical type and the solitary type [12].The classical form may present at birth or manifest before 20 years of age. The lesions in a classical form commonly occur over the pelvic girdle, lower back, gluteal region, and thighs [13]. Lesions have also been documented at less common locations such as the neck [14], perianal region [2], nose [15], groin [16], and pinna [17]. The lesions develop slowly but might grow large in size if left untreated, as seen in the current example. The second clinical form is the solitary type, which can be misdiagnosed as a skin tag. The solitary form most commonly occurs around the fifth decade of life and can form anywhere on the body [18]. The solitary form has also been documented in unusual locations such as eyelids, nose, scalp, and clitoris [4].

Clinically, nevus sebaceous, connective tissue nevus, neurofibroma, lymphangioma, hemangioma, focal dermal hypoplasia (Goltz syndrome), neurofibroma, acrochordons (fibroepithelial polyp), melanocytic nevus, lipoma, and lipoblastoma were kept as differential diagnoses [19]. The differentiating process is aided by histopathological testing [20]. Although most cases of NLCS show no symptoms, rare cases can have aberrant growth or shape, including giant NLCS, comedo-like plugs, foul-smelling discharges, and ulcerated lesions after external trauma or ischemia [21].

Despite several hypotheses, the pathophysiology of pedunculated NLCS is unclear. The proposed explanations include the displacement of subcutaneous adipose tissue into the dermis, degenerative changes in the collagen and elastic tissue, and origination and differentiation from the walls of dermal vessels [12]. The lesions gradually increase in size but stabilize over time. The lesions in the current case ranged in size from 0.5 cm to the largest being 6 cm x 3 cm [22].

Various therapies have been implicated in treating NLCS, which include surgical resection, electrocautery, cryotherapy, phosphatidylcholine injection, and CO2 laser. Nevus lipomatosus cutaneous superficialis is frequently asymptomatic; therefore, the primary line of therapy is surgical resection with primary wound closure [23]. Individuals who do not want to undergo surgery might go for cryotherapy, which is its only indication, and the results are partially effective [13]. Various treatment options have been discovered and tried with successful results. It has also been reported that CO2 laser therapy can cause recurrence, and therefore physicians should take note of it [24]. Intralesional phosphatidylcholine and sodium deoxycholate have also been tried as possibly effective and tolerable treatments for classic NLS. The combined approach of electrosurgery and conventional excisional surgery aids in the complete removal of large NLCS lesions, minimizing recurrence and promoting effective healing. This method also supports improved recovery by addressing both functional and cosmetic concerns, enhancing patient satisfaction [25].

## Conclusions

This case emphasizes the critical importance of a conservative and staged approach in managing NLCS, particularly for larger lesions. Serial excision was key in gradually removing the lesion while minimizing scarring and preserving the surrounding tissue. By opting for a staged removal, the procedure reduced trauma to the skin, minimized complications, and allowed the tissue to heal effectively between surgeries. This approach led to an excellent cosmetic result, with only a small hypopigmented patch remaining at the site after six months. For patients with extensive lesions, this method ensures better functional and aesthetic outcomes, making it an effective strategy in cosmetically sensitive areas where large scars could have a significant impact on the patient’s appearance and quality of life. Early diagnosis and management are crucial to prevent morbidity and significant disfigurement.

Nevus lipomatosus cutaneous superficialis, while rare, can easily be overlooked or misdiagnosed, making clinical awareness essential for timely intervention. In this case, histopathological confirmation guided the treatment approach, with surgical excision being the most effective option, particularly for the classical form of NLCS. Understanding the nature of the condition and tailoring treatment to the size, location, and extent of the lesions ensures that the best outcomes are achieved. Offering patients a staged, conservative approach, especially for large lesions, allows for both optimal cosmetic results and minimized risks, leading to greater patient satisfaction and improved long-term prognosis.
